# Supplementary figures and images for: The V-ATPase–ATG16L1 axis recruits LRRK2 to facilitate the lysosomal stress response
Source: J Cell Biol. 2024 Jan 16;223(3):e202302067. doi: 10.1083/jcb.202302067 (PMC10791558; doi:10.1083/jcb.202302067)

Fig. 2A

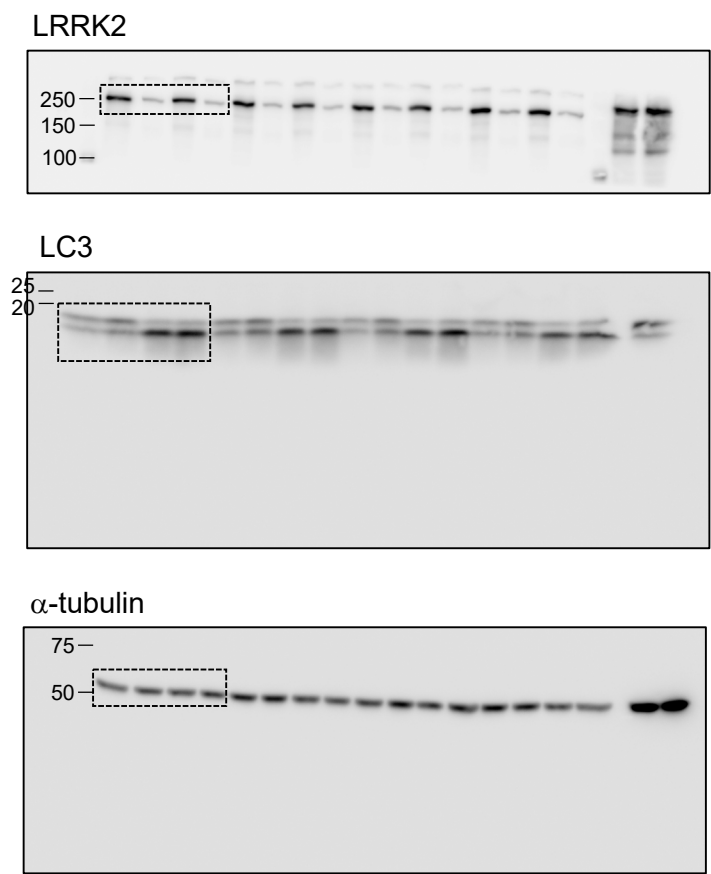

Supplement: SourceData F2 — is the source file for Fig. 2. [file JCB_202302067_SourceDataF2.pdf]

**Fig. 4A**

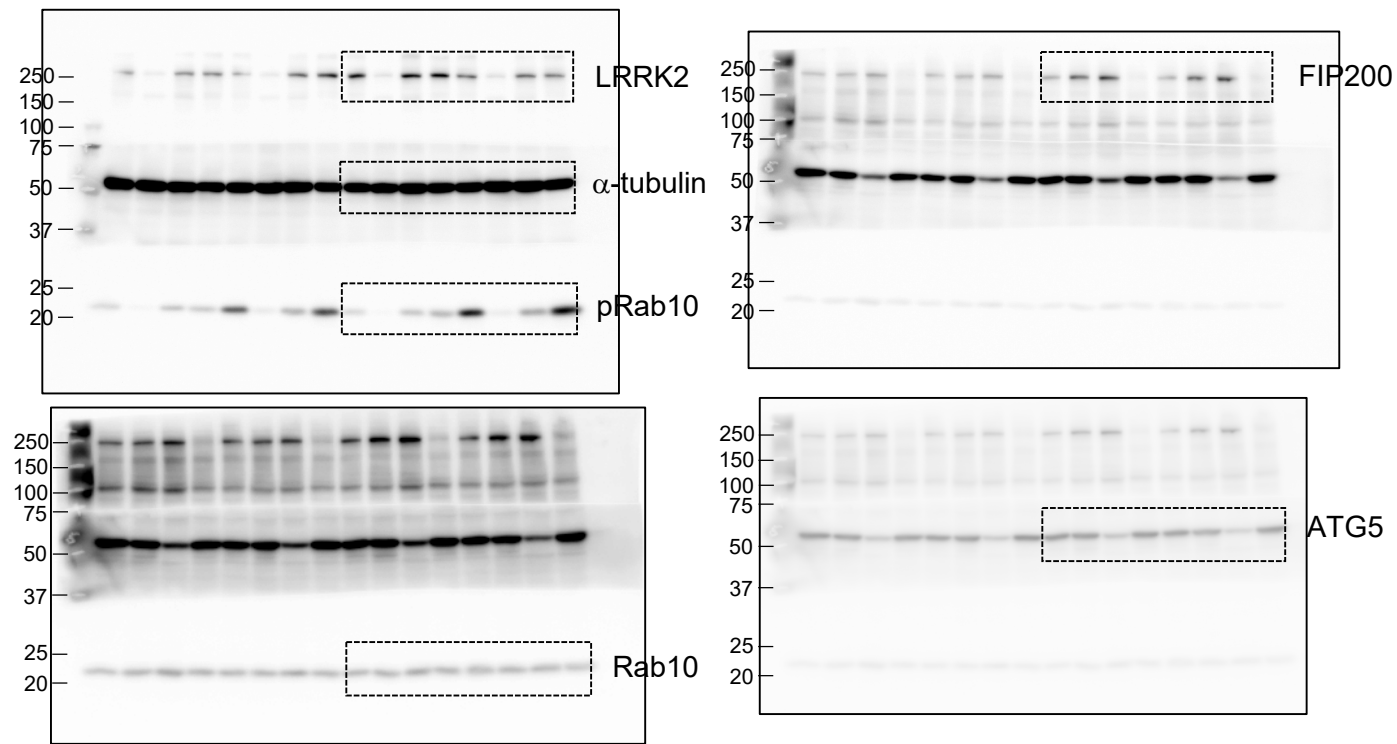

**Fig. 4C**

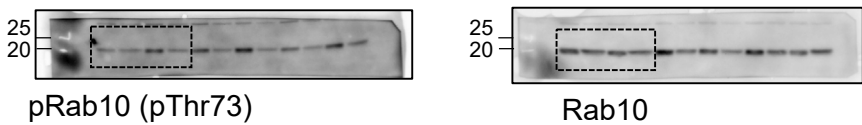

**Fig. 4E**

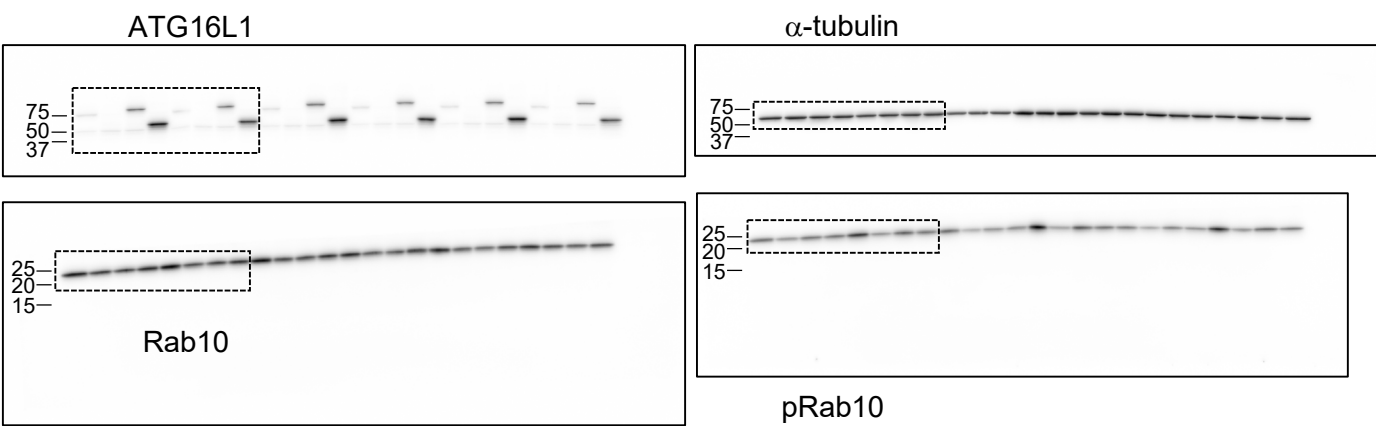

Supplement: SourceData F4 — is the source file for Fig. 4. [file JCB_202302067_SourceDataF4.pdf]

**Fig. 5A**

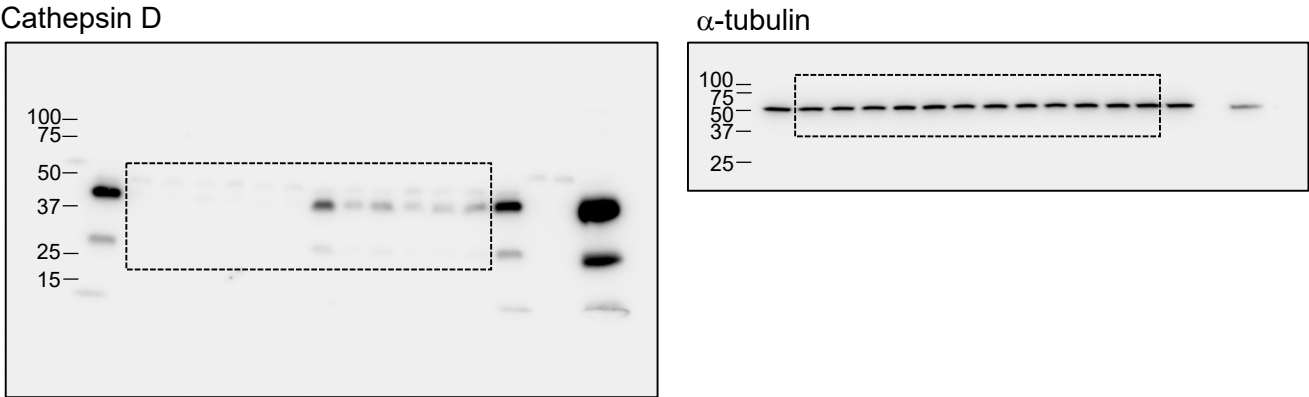

**Fig. 5C**

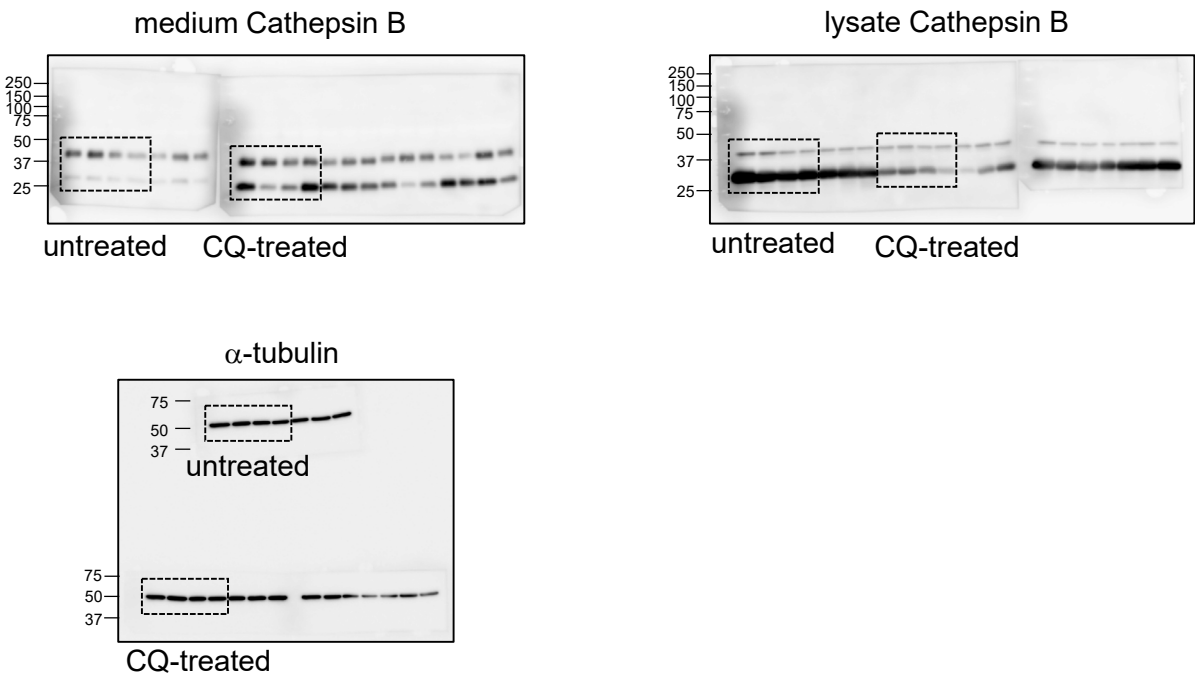

Supplement: SourceData F5 — is the source file for Fig. 5. [file JCB_202302067_SourceDataF5.pdf]

**Fig. S2B**

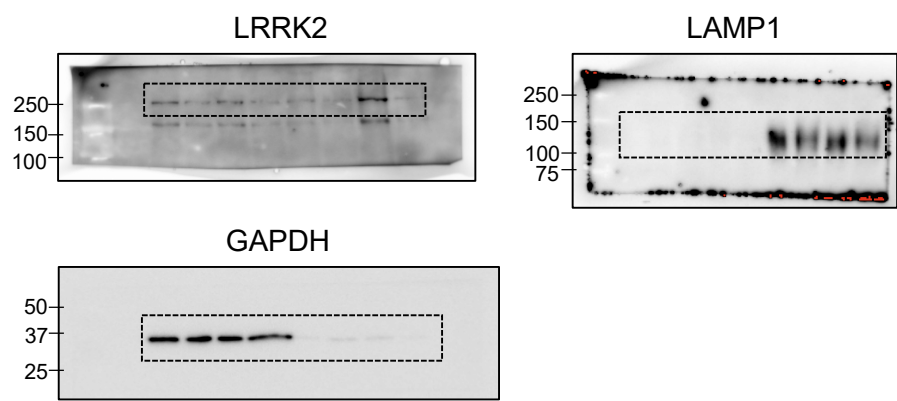

Supplement: SourceData FS2 — is the source file for Fig. S2. [file JCB_202302067_SourceDataFS2.pdf]

**Fig. S3C**

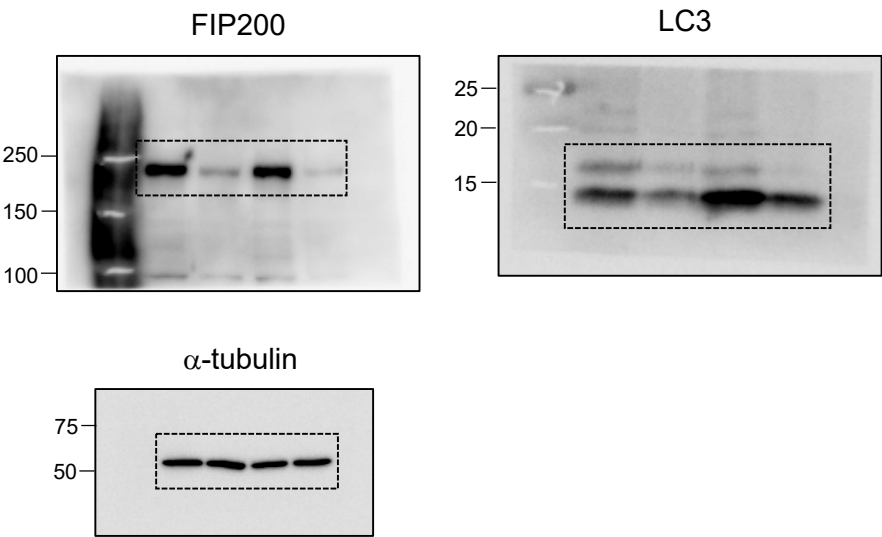

**Fig. S3D**

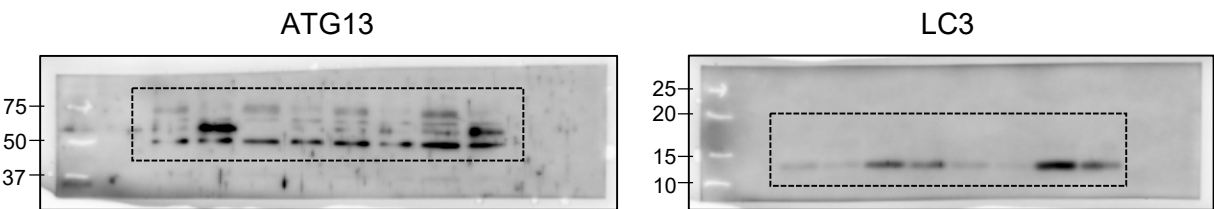

Supplement: SourceData FS3 — is the source file for Fig. S3. [file JCB_202302067_SourceDataFS3.pdf]

**Fig. S4A**

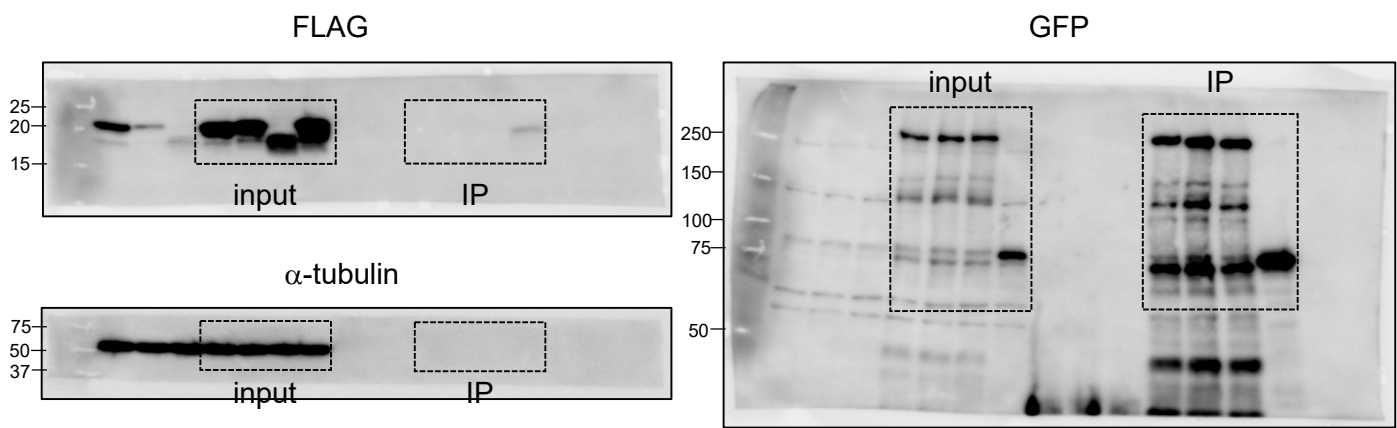

**Fig. S4B**

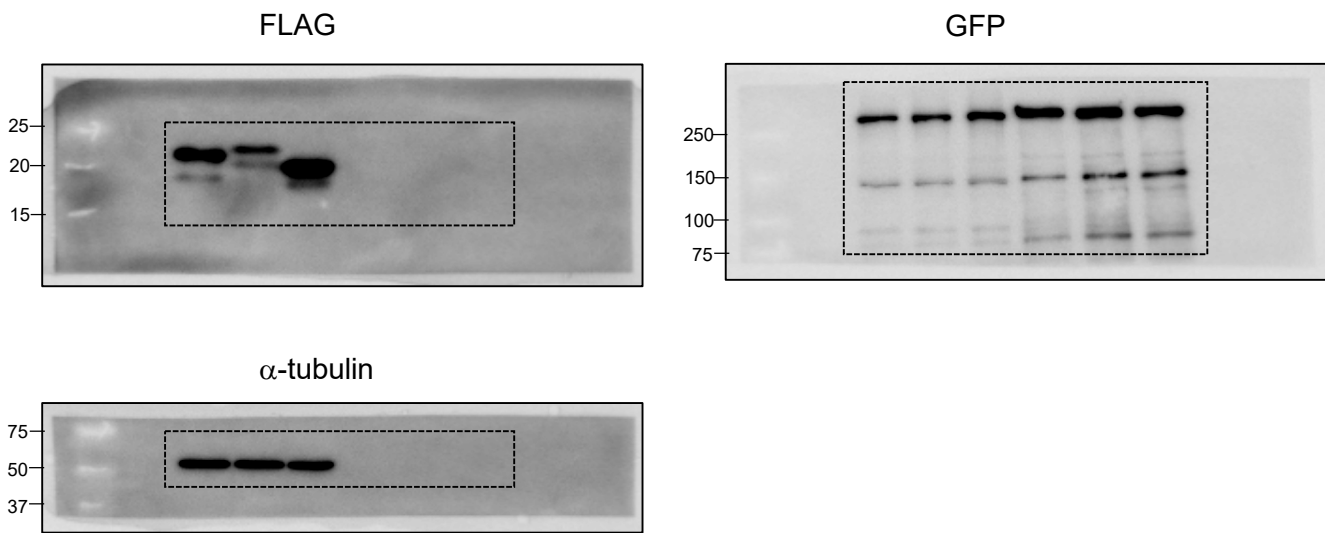

Supplement: SourceData FS4 — is the source file for Fig. S4. [file JCB_202302067_SourceDataFS4.pdf]
